# Single Gene Prognostic Biomarkers in Ovarian Cancer: A Meta-Analysis

**DOI:** 10.1371/journal.pone.0149183

**Published:** 2016-02-17

**Authors:** Scooter Willis, Victor M. Villalobos, Olivier Gevaert, Mark Abramovitz, Casey Williams, Branimir I. Sikic, Brian Leyland-Jones

**Affiliations:** 1 Dept. of Molecular and Experimental Medicine, Avera Cancer Institute, Sioux Falls, SD, United States of America; 2 Division of Medical Oncology, UC Denver, Denver, CO, United States of America; 3 Stanford University, Stanford, CA, United States of America; University of North Carolina School of Medicine, UNITED STATES

## Abstract

**Purpose:**

To discover novel prognostic biomarkers in ovarian serous carcinomas.

**Methods:**

A meta-analysis of all single genes probes in the TCGA and HAS ovarian cohorts was performed to identify possible biomarkers using Cox regression as a continuous variable for overall survival. Genes were ranked by p-value using Stouffer’s method and selected for statistical significance with a false discovery rate (FDR) <.05 using the Benjamini-Hochberg method.

**Results:**

Twelve genes with high mRNA expression were prognostic of poor outcome with an FDR <.05 (AXL, APC, RAB11FIP5, C19orf2, CYBRD1, PINK1, LRRN3, AQP1, DES, XRCC4, BCHE, and ASAP3). Twenty genes with low mRNA expression were prognostic of poor outcome with an FDR <.05 (LRIG1, SLC33A1, NUCB2, POLD3, ESR2, GOLPH3, XBP1, PAXIP1, CYB561, POLA2, CDH1, GMNN, SLC37A4, FAM174B, AGR2, SDR39U1, MAGT1, GJB1, SDF2L1, and C9orf82).

**Conclusion:**

A meta-analysis of all single genes identified thirty-two candidate biomarkers for their possible role in ovarian serous carcinoma. These genes can provide insight into the drivers or regulators of ovarian cancer and should be evaluated in future studies. Genes with high expression indicating poor outcome are possible therapeutic targets with known antagonists or inhibitors. Additionally, the genes could be combined into a prognostic multi-gene signature and tested in future ovarian cohorts.

## Introduction

Ovarian cancer is the fifth leading cause of cancer-related deaths with an estimated 22,000 new cases a year and 15,000 deaths in the United States [1]. From 1950–2008, the ovarian cancer death rate of 10 per 100,000 women has remained unchanged, indicating the need to identify new and novel therapies for this disease. Standard of care for advanced-stage ovarian cancer is extensive debulking surgery followed by chemotherapy [2–4]. A significant factor in the elevated mortality rate is the lack of disease-specific symptoms resulting in late-stage diagnoses where the cure rate for early-stage diagnoses is 90% [5,6]. Identification of serum-based biomarkers and imaging to detect early-stage ovarian cancer for routine screening is one potential strategy to improve overall survival (OS) [7].

Various groups have identified large multi-gene signatures that were prognostic of outcome in molecularly profiled ovarian tumor samples [8–21]. We sought to identify single-gene prognostic biomarkers using meta-analysis of publicly available mRNA expression data from ovarian cohorts with known drug-gene interactions that could be potentially used to indicate alternative treatment strategies.

## Materials and Methods

### Meta-Analysis

Data extraction was conducted in agreement with the Preferred Reporting Items for Systematic Reviews and Meta-Analyses (PRISMA) guidance (S1 File) [22]. The protocol used to perform this meta-analysis was not registered prior given that we are using data as published and a Cox regression analysis as a continuous variable without any pre-determined cutoffs. We used Cox regression analysis to determine the Wald Test p-value for each Affymetrix probe as a continuous variable where mRNA expression is represented as a z-score. The Cox proportional hazards model was used to calculate the hazard ratios (HR) for OS and their 95% confidence intervals (CI) for each probe. The p-value for each single probe from each cohort was combined using Stouffer’s method to combine the results from two independent ovarian cohorts. The resulting p-value for each probe in the combined cohorts was used to rank the prognostic probes. Probes with a false discovery rate (FDR) <.05 using the Benjamini-Hochberg method were selected as being statistically significant. For Cox regression survival analysis and Kaplan–Meier figures, the Biojava3-survival module from BioJava [23] was used. The Biojava3-survival module is a direct port of the Cox regression C code in the R survival package [24,25].

### Meta-Analysis Cohorts

The TCGA Ovarian HG-U133A cohort was downloaded on May 21, 2015 from the Broad Institute FireBrowse Data Portal (www.firebrowse.org). This TCGA cohort was used as the discovery cohort consisting of 470 samples with 249 events for OS. The OS events were determined from the metadata “vital_status” and the event/censor time was the maximum time from “days_to_last_followup” and “days_to_death” provided in OV.clin.merged.picked.txt. Additional metadata was merged from OV.clin.merged.txt. The TCGA ovarian cohort consists of 77% stage III and 15% stage IV serous carcinoma patients.

Next, a collection of ovarian data sets was downloaded on December 6, 2013 from the kmplot.com website consisting of 1,287 samples [26] and was used as the second cohort in the meta-analysis. The ovarian cohort used for outcome analysis at the kmplot web site is a collection of published cohorts profiled on the Affymetrix platform where the raw CEL files were available for MAS5 normalization as a combined cohort and unique sample identification. The HAS ovarian cohort (HAS = Hungarian Academy of Sciences) includes the TCGA ovarian cohort and those samples were removed to establish an independent cohort. Additionally, the HAS ovarian cohort contains a high number of stage I and stage II samples that were removed to match the high number of stage III and stage IV samples in the TCGA ovarian cohort. The resulting independent HAS ovarian validation cohort consisted of 313 samples with 167 events for OS (91% stage III and 9% stage IV). The metadata for HAS ovarian validation cohort indicates 188 serous carcinoma, 6 endometrial and 121 undefined samples. The HAS ovarian cohort includes samples of seven independent cohorts GSE14764, GSE15622, GSE19829, GSE3149, GSE9891, GSE18520 and GSE26712. The HAS ovarian metadata is limited and does not indicate patient age or other standard cohort metrics.

The TCGA Ovarian Cohort and HAS Cohort are well known publicly available cohorts that can be downloaded by researchers for meta-analysis. The co-authors have no affiliation with the ovarian cohorts and no changes were made to mRNA expression values used in the meta-analysis.

### Enrichment Analysis

Gene-annotation enrichment analysis was performed using DAVID tools using default settings [27].

## Results

The results of the meta-analysis for statistically significant genes with an FDR <.05 where high expression indicates poor outcome can be found in Table 1, and where low expression indicates poor outcome can be found in Table 2. In total, each of the 17,169 Affymetrix probes were used to determine a prognostic p-value using cox regression analysis. The p-values for each probe in two independent cohorts were combined using Stouffer’s method and the probes ranked. The 17,169 probes were used to determine the FDR where probes with an FDR <.05 were considered statistically significant. In total, 32 probes had an FDR <.05 where 12 had high expression indicating poor outcome and 20 had low expression indicating poor outcome. Genes with high expression indicating poor outcome are possible therapeutic targets with known antagonists or inhibitors.

**Table 1:** Probes where high expression is prognostic of poor outcome with an FDR <0.05. (25–75)% is the difference in expression of the 25^th^ and 75^th^ percentile expression on a log scale. The Stouffer p-value was used as the ranking metric combining the p-values from each cohort.

		TCGA Ovarian Broad OS Stage 3 and 4			HAS Ovarian OS Stage 3 and 4(No TCGA)				
REF	Probe	p-value	HR 95% CI	(25–75)%	p-value	HR 95% CI	(25–75)%	Stouffer	FDR
AXL	202686_s_at	2.29E-04	1.27 CI(1.12–1.45)	1.3	0.001	1.29 CI(1.10–1.50)	0.7	1.83E-06	0.022
APC	203525_s_at	4.92E-05	1.33 CI(1.16–1.52)	0.8	0.017	1.22 CI(1.04–1.43)	0.7	5.01E-06	0.029
RAB11FIP5	210879_s_at	7.59E-05	1.29 CI(1.14–1.46)	0.7	0.039	1.19 CI(1.01–1.40)	0.4	1.82E-05	0.041
C19orf2	211563_s_at	0.007	1.19 CI(1.05–1.35)	1.1	1.85E-04	1.36 CI(1.16–1.60)	0.6	2.92E-05	0.041
CYBRD1	217889_s_at	3.91E-04	1.24 CI(1.10–1.40)	2	0.014	1.21 CI(1.04–1.41)	1.3	2.99E-05	0.041
PINK1	209019_s_at	0.006	1.19 CI(1.05–1.34)	0.7	4.83E-04	1.31 CI(1.12–1.52)	0.5	4.42E-05	0.041
LRRN3	209840_s_at	4.84E-05	1.21 CI(1.10–1.32)	0.3	0.118	1.13 CI(0.97–1.33)	1.8	4.78E-05	0.041
AQP1	207542_s_at	0.005	1.19 CI(1.05–1.35)	0.8	8.19E-04	1.33 CI(1.12–1.57)	0.7	5.02E-05	0.041
DES	214027_x_at	0.005	1.18 CI(1.05–1.32)	0.5	8.46E-04	1.29 CI(1.11–1.49)	1.3	5.13E-05	0.041
XRCC4	205072_s_at	0.053	1.13 CI(1.00–1.27)	0.6	3.62E-06	1.48 CI(1.26–1.75)	0.7	6.35E-05	0.047
BCHE	205433_at	4.09E-04	1.23 CI(1.10–1.37)	0.7	0.033	1.20 CI(1.01–1.43)	1.7	7.10E-05	0.048
ASAP3	219103_at	1.26E-04	1.27 CI(1.13–1.44)	0.6	0.088	1.14 CI(0.98–1.32)	0.9	7.34E-05	0.048

**Table 2:** Probes where low expression is prognostic of poor outcome with an FDR <0.05. (25–75)% is the difference in expression of the 25^th^ and 75^th^ percentile expression on a log scale. The Stouffer p-value was the ranking metric combining the p-values from each cohort.

		TCGA Ovarian Broad OS Stage 3 and 4			HAS Ovarian OS Stage 3 and 4(No TCGA)				
REF	Probe	p-value	HR 95% CI	(25–75)%	p-value	HR 95% CI	(25–75)%	Stouffer	FDR
LRIG1	211596_s_at	1.33E-04	0.79 CI(0.69–0.89)	1.5	0.003	0.79 CI(0.67–0.92)	1.3	2.58E-06	0.022
SLC33A1	203164_at	1.39E-04	0.79 CI(0.70–0.89)	0.9	0.009	0.83 CI(0.71–0.95)	0.5	7.23E-06	0.030
NUCB2	203675_at	1.52E-04	0.79 CI(0.69–0.89)	1.1	0.01	0.82 CI(0.70–0.95)	0.6	8.71E-06	0.030
POLD3	212836_at	0.017	0.86 CI(0.76–0.97)	0.6	3.53E-06	0.67 CI(0.56–0.79)	0.5	1.05E-05	0.030
ESR2	211120_x_at	1.20E-04	0.77 CI(0.67–0.88)	0.2	0.038	0.86 CI(0.74–0.99)	1.1	2.67E-05	0.041
GOLPH3	217803_at	4.34E-04	0.80 CI(0.71–0.91)	0.6	0.014	0.83 CI(0.72–0.96)	0.5	3.31E-05	0.041
XBP1	200670_at	0.006	0.84 CI(0.74–0.95)	1.2	3.72E-04	0.76 CI(0.65–0.88)	0.8	3.74E-05	0.041
PAXIP1	212825_at	0.008	0.85 CI(0.75–0.96)	0.8	2.22E-04	0.76 CI(0.66–0.88)	0.5	3.88E-05	0.041
CYB561	217200_x_at	0.004	0.82 CI(0.72–0.94)	0.7	8.93E-04	0.76 CI(0.65–0.89)	0.9	4.09E-05	0.041
POLA2	204441_s_at	0.036	0.87 CI(0.77–0.99)	0.7	5.44E-06	0.72 CI(0.63–0.83)	0.7	4.15E-05	0.041
CDH1	201131_s_at	0.004	0.83 CI(0.73–0.94)	0.8	9.13E-04	0.79 CI(0.69–0.91)	0.8	4.16E-05	0.041
GMNN	218350_s_at	0.014	0.86 CI(0.77–0.97)	1.1	1.05E-04	0.74 CI(0.63–0.86)	0.8	5.15E-05	0.041
SLC37A4	217289_s_at	5.79E-04	0.81 CI(0.72–0.91)	0.4	0.017	0.81 CI(0.69–0.96)	0.9	5.24E-05	0.041
FAM174B	51158_at	0.006	0.82 CI(0.71–0.95)	0.9	0.001	0.78 CI(0.68–0.91)	0.9	7.12E-05	0.048
AGR2	209173_at	0.014	0.85 CI(0.74–0.97)	2.6	2.05E-04	0.74 CI(0.63–0.87)	2.7	7.61E-05	0.048
SDR39U1	213398_s_at	0.008	0.84 CI(0.74–0.96)	0.7	6.92E-04	0.77 CI(0.66–0.89)	0.5	7.92E-05	0.048
MAGT1	221553_at	5.05E-04	0.80 CI(0.70–0.91)	0.9	0.031	0.85 CI(0.74–0.99)	0.8	8.13E-05	0.048
GJB1	204973_at	0.002	0.81 CI(0.71–0.92)	1.2	0.007	0.83 CI(0.72–0.95)	1.5	8.58E-05	0.049
SDF2L1	218681_s_at	0.001	0.81 CI(0.72–0.92)	1.1	0.017	0.83 CI(0.72–0.97)	0.7	8.94E-05	0.050
C9orf82	219276_x_at	0.004	0.86 CI(0.78–0.95)	0.8	0.003	0.81 CI(0.71–0.93)	0.6	9.56E-05	0.051

The complete list of probes and resulting p-values are provided in the supplemental. For the probes with an FDR <.05 all HR directions were in agreement in the two cohorts providing further support that the single probes were valid biomarkers with minimal false positives. The expectation is that a valid biomarker would have a consistent prognostic HR in that high expression in both cohorts would denote poor outcome. If a statistically significant cutoff for Stouffer’s p-value <.001 without an FDR correction was used, it resulted in an additional 105 probes, where 8 (7.6%) of the probes did not have HR agreement in the two cohorts and would be considered false positives. Using a Stouffer p-value <.01 identified an additional 432 probes where 70 (16%) of the probes did not have HR agreement. Using an FDR cutoff of <.05 established a list of 32 probes that were informative of outcome.

Gene enrichment analysis of the 20 genes where low expression indicates poor prognosis were associated with endoplasmic reticulum with a Benjamin correction p-value <.05. For the 12 genes where high expression indicates poor prognosis no statistically significant association.

## Discussion

The use of meta-analysis of existing data in publicly available ovarian cancer cohots may yield genes that should be investigated more closely and that may eventually lead to new drug treatments for ovarian cancer patients that have been slow in coming. Chemotherapy is currently used as the standard of care in conjunction with debulking surgery in patients with advanced ovarian cancer [2–4]. The addition of targeted therapy in combination with chemotherapy may improve OS, however, identification of these types of drugs remains elusive. Genes that are overexpressed in ovarian tumors are not only potential biomarkers of prognosis but may also be therapeutic targets if those genes correlate with a poor outcome. Conversely, overexpressed genes that are associated with a good outcome can be unintentionally targeted by standard cancer treatments or off-target effects from drugs the patients may be taking for other health issues. We conducted a meta-analysis of mRNA expression data from two ovarian cohorts and used various statistical tools to identify 12 overexpressed (Table 1) and 20 under-expressed (Table 2) genes that correlated with a poor outcome.

In this study, overexpression of 12 genes and underexpression of 20 genes were associated with a poor outcome. Thus, our meta-analysis has implicated genes that may be prognostic as well as potential therapeutic targets to pursue in the treatment of ovarian cancer. The ability to generate single gene lists from published ovarian cohorts could also lead to a more thorough understanding of what genes contribute to the ovarian cancer tumorigenic process. The use of bioinformatics, therefore, in conjunction with analysis of clinical and literature databases will be required to cull these gene lists in order to focus on the most potentially relevant ones.

## Supporting Information

S1 File PRISMA Checklist. (DOC)
